# Quantitative estimation of soil salinity by means of different modeling methods and visible-near infrared (VIS–NIR) spectroscopy, Ebinur Lake Wetland, Northwest China

**DOI:** 10.7717/peerj.4703

**Published:** 2018-05-03

**Authors:** Jingzhe Wang, Jianli Ding, Aerzuna Abulimiti, Lianghong Cai

**Affiliations:** College of Resources and Environment Science, Xinjiang University, Urumqi, Xinjiang, China; Key Laboratory of Smart City and Environment Modelling of Higher Education Institute, Xinjiang University, Urumqi, Xinjiang, China; Key Laboratory of Oasis Ecology, Xinjiang University, Urumqi, Xinjiang, China

**Keywords:** Ebinur Lake, RF, VIS–NIR, PLSR, Soil salinity, Machine learning, Wetland

## Abstract

Soil salinization is one of the most common forms of land degradation. The detection and assessment of soil salinity is critical for the prevention of environmental deterioration especially in arid and semi-arid areas. This study introduced the fractional derivative in the pretreatment of visible and near infrared (VIS–NIR) spectroscopy. The soil samples (*n* = 400) collected from the Ebinur Lake Wetland, Xinjiang Uyghur Autonomous Region (XUAR), China, were used as the dataset. After measuring the spectral reflectance and salinity in the laboratory, the raw spectral reflectance was preprocessed by means of the absorbance and the fractional derivative order in the range of 0.0–2.0 order with an interval of 0.1. Two different modeling methods, namely, partial least squares regression (PLSR) and random forest (RF) with preprocessed reflectance were used for quantifying soil salinity. The results showed that more spectral characteristics were refined for the spectrum reflectance treated via fractional derivative. The validation accuracies showed that RF models performed better than those of PLSR. The most effective model was established based on RF with the 1.5 order derivative of absorbance with the optimal values of *R*^2^ (0.93), RMSE (4.57 dS m^−1^), and RPD (2.78 ≥ 2.50). The developed RF model was stable and accurate in the application of spectral reflectance for determining the soil salinity of the Ebinur Lake wetland. The pretreatment of fractional derivative could be useful for monitoring multiple soil parameters with higher accuracy, which could effectively help to analyze the soil salinity.

## Introduction

Soil salinization is one of the most common forms and drivers of land degradation, and entails significant environmental, social, and economic consequences, especially in arid and semi-arid areas (Akramkhanov et al., 2011; Ding & Yu, 2014; Nawar, Buddenbaum & Hill, 2015). It is estimated that 15% of the total land area of China is affected by salinity (Peng et al., 2016; Wang et al., 2007). Oasis ecosystem is the material and ecological base of arid and semi-arid areas (Abliz et al., 2016). With the rapidly increasing population densities and drastic land use changes over the past few decades, soil salinization has become the main restraint not only for a sustainable development of oasis agriculture, but also for the stability of regional ecosystems (Scudiero, Skaggs & Corwin, 2014). Timely detection as well as assessment of soil salinity are essential to regional ecological stability, and these problems have attracted considerable attention worldwide in recent years.

Traditionally, the detection and assessment of soil salinity require intensive field-derived work, e.g., the electromagnetic measurements of soil electrical conductivity (EC) or time-consuming laboratory experiments (Ding & Yu, 2014). *In-situ* measurements have been widely proved to be the most valid approach to assess soil salinity; however, they could only provide limited point information, rather than large-scale spatial global information (Dehaan & Taylor, 2002). Compared to conventional laboratory analysis methods, the remote sensing technology is a promising alternative approach for quantitative evaluation of soil attributes due to its obvious characteristics, including rapid response, low cost, wide view filed, and fast acquisition (Ben-Dor & Banin, 1995; Farifteh, Farshad & George, 2006; Metternicht & Zinck, 2003). Remote sensing data is well-adopted for mapping and assessing various characteristics of surface soil across different scale (Allbed, Kumar & Aldakheel, 2014; Corwin et al., 2003). Therefore, based on the different spectral reflection and absorption characteristics of the VIS–NIR bands to soil salinity, spectral analysis technology could be an alternative to ensure accurate estimation of salt content in soils. (Cécillon et al., 2009; Islam, Singh & McBratney, 2003).

The applicability of VIS–NIR has been investigated and the results showed that the characteristic bands cover the absorption spectra of NaCl (1,930 nm), KCl (1,430 nm), and MgSO_4_ (1,480 nm) (Cécillon et al., 2009; Stenberg et al., 2010). The different spectral reflection and absorption characteristics of the VIS–NIR bands to soil salinity laid the foundation of quantifying soil salinity. The partial least squares regression (PLSR) and artificial neural network (ANN) have been successfully used for predicting main salt concentrations of soils using reflectance spectroscopy (Farifteh et al., 2007). Using raw reflectance and pretreatment by Savitzky–Golay (S-G) smoothing, first derivative (FD) and second derivative (SD), the performance of PLSR, and multivariate adaptive regression splines (MARS) were compared to identify the best regression approach to quantify soil salinity (Nawar, Buddenbaum & Hill, 2015). Viscarra Rossel & Behrens (2010) compared the performance of PLSR, ANN, random forest (RF) and five other different data mining algorithms for the assessment of organic carbon (OC), clay content and pH of soil. Because it can consider dimension synthesis and solve the multiple collinearity problems among independent variables, PLSR is a frequently used and reliable linear regression method especially for quantitative research (Llndber, Persson & Wold, 1983; Wold, Sjöström & Eriksson, 2001). This technology has proved to be capable of inference capabilities, which could simulate the potential linear relationship between some specific soil attributes and corresponding VIS–NIR reflectance (Farifteh et al., 2007; Nawar et al., 2014).

However, the non-uniform data distribution and non-linear reflectance behavior indicate that the application of PLSR is insufficient, which has some limitations (Nawar et al., 2016). The RF is an ensemble machine learning technique with the capability of solving classification, regression, and other tasks in different fields (Breiman, 2001). Differing from existing linear and non-linear regression modeling methods, RF has acceptable predicting performance even if most independent variables are noise (Chen & Liu, 2005). Owning to its higher quality implementations, fewer restrictions and excellent performance, RF has been widely used in bioinformatics, hyperspectral data classification and other related disciplines, and generally exhibits higher accuracy and efficiency (Díaz-Uriarte & Alvarez de Andrés, 2006; Pal, 2005; Rodriguez-Galiano et al., 2012; Shi & Horvath, 2006; Sun & Schulz, 2015). Numerous studies have demonstrated that RF provided better spectral estimations than those by PLSR (Clark, Roberts & Clark, 2005; Douglas et al., 2018; Stevens et al., 2013; Viscarra Rossel & Behrens, 2010). As a source of high-dimensional data, spectral reflectance data possess high spectral resolution, consecutive wavebands, and a variety spectral information (Wang et al., 2017b). Quantifying soil salinity with VIS–NIR reflectance is therefore challenging, due to the large amount of irrelevant spectral data and inherent noise. Furthermore, a defect of signal-to-noise ratio decreasing at longer wavelengths might affect the deep application from VIS–NIR spectroscopy. In the study of estimating soil parameters reported previously, spectral reflectance has been applied directly, and the relationship between integer derivative transforms (FD and SD) of spectral data and the salt content or EC of soils has been well studied (Nawar, Buddenbaum & Hill, 2015; Shi et al., 2013; Viscarra Rossel & Webster, 2012). However, the detection of spectral information via FD and SD with wider order intervals could, to some degree, result in the loss of spectral information. Some studies have demonstrated significant improvements on potential applications of the fractional derivative in various fields (Chen, 2008; Wang et al., 2017a; Wang et al., 2017c; Zhang et al., 2016a). With the narrower order interval, the fractional derivative expanded the theoretical concept of classic derivative. It has proved to be an effective pretreatment of spectral data (Wang et al., 2017b; Zhang et al., 2016a). Moreover, the algorithm has been used for preprocessing the spectral data of soils, and the results demonstrated that it could improve the sensitivity between the dependent and independent variables in the spectral analysis (Xia et al., 2017).

Although some existing researches have estimated local soil salinity and clay content using VIS–NIR preprocessed by fractional derivative, accurate and stable fractional order for the ideal estimation have not been implemented yet (Wang et al., 2017b; Zhang et al., 2016a). Substantial efforts in predicting soil salinity suffer from the limitations of different modeling approaches to provide a generalized model over various scales and datasets. This study aimed to fill the gap and to advance the use of VIS–NIR for quantifying soil salinity based on the pretreatment of fractional derivative. The main objectives of this study were (1) to establish a generalized stable model to predict soil salinity by means of VIS–NIR spectroscopy; (2) to select the optimal fractional derivative order for soil salinity estimation; (3) to compare linear (PLSR) and non-linear (RF) models for the most effective quantitative prediction of soil salinity.

## Materials and Methods

### Study area

The Ebinur Lake wetland, a core area of Oasis–Desert System in Central Asia, was selected as the study area. Ebinur Lake is located in the south-western region of the Junggar Basin (44°20′ ∼45°29′N, 82°06′ ∼83°40′E, northwestern XUAR) (He et al., 2015; Liu et al., 2011). The total area of the study area is 2,670.8 km^2^ (Ge et al., 2016). The wetland has a typical temperate continental climate with scarce precipitation (100–200 mm), strong potential evaporation (≥1,600 mm) and strong winds (≥8 m/s, on 164 days) annually. The soil salinity of the study area varies from very slightly saline to strongly saline and local prevalent salt minerals are NaCl (Liu et al., 2011). According to the World Reference Base for Soil Resources (WRB), local prevalent soil types are mainly Arenosols, Solonetz, and Solonchaks (Deckers et al., 2002; He et al., 2015). The study area is characterized by fragile ecology and is particularly sensitive to climate change and human activities. In recent years, the drawdown of dry lakebed (playa) has exposed broad hard salt crusts and saline desert, which might have a range of negative effects on the local fragile environment (Liu et al., 2011). To protect the important wetland ecosystems in arid areas, the Chinese government has designated the adjoining of the Ebinur Lake wetland as a National Nature Reserve in April 2007 (Zhang et al., 2016b).

### Sample collection and chemical analysis

**Figure 1:**
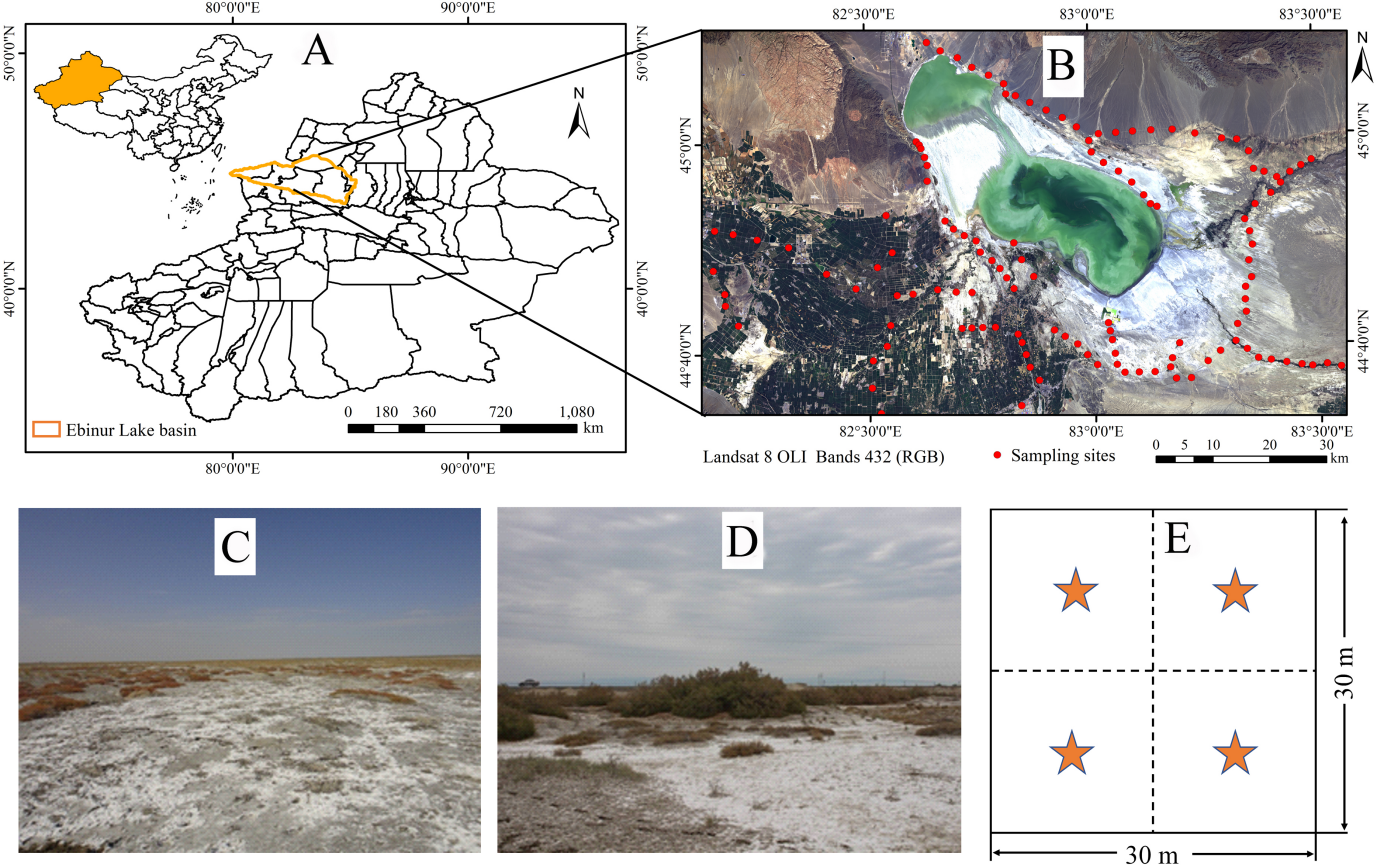
Distribution of sampling sites in the study area. (A) Location map of XUAR. (B) Ebinur Lake wetland region. (C and D) Typical landscape photograph (Photograph credit: Jingzhe Wang). (E) The schema of sampling method (4 points) within the 30 m × 30 m cell grid.

To ensure the relative representative and homogeneous soil types, soil texture and landscape, the samples were obtained from a total of 100 sampling units on a grid of 30 m × 30 m (because the spatial resolution of Landsat satellite imagery is 30 m) throughout the study area in October 2016 (Fig. 1). A portable GPS meter (Garmin GPS 72) was employed to record the coordinates of each sampling point, as displayed in Fig. 1E. In each unit, about 0.50 kg of topsoil from depths of 0 to 5 cm was collected at four randomly selected sampling sites. Each sample was placed into a sealed watertight bag and labeled. A total of (4 × 100) topsoil samples were obtained and preserved for the soil attributes measurements. All samples were sufficiently air-dried (over 35 °C) for two weeks, ground, and then passed through a 2.0 mm sieve to wipe off plant materials, residue, and stones. Prior to chemical analysis, organic carbon (OC) was removed using hydrogen peroxide (H_2_O_2_, 30%). We determined the soil salinity and pH value with a digital multiparameter measuring apparatus (Multi 3420 Set B, WTW GmbH, Germany) equipped with the composite electrode (TetraCon 925 and SenTix 940) in a 1:5 soil-water extraction solution at room temperature (25 °C). The measurement of soil particle size was conducted using a particle analyzer system (Bluewave S3500, Largo, FL, USA). Seven main soluble ions concentrations, i.e., potassium (K^+^), sodium (Na^+^), calcium ion (Ca^2+^), magnesium ion (Mg^2+^), chloridion (Cl^−^), sulphane (SO${}_{4}^{2-}$) and mbicarbonate (HCO${}_{3}^{-}$) were also evaluated according to the standardized method outlined by Bao (2000). The concentrations of K^+^ and Na^+^ were measured using flame photometry method; Ca^2+^ and Mg^2+^ were measured using EDTA complexometric titration method; Cl^−^ was determined using the silver nitrate (AgNO3) titration method; SO${}_{4}^{2-}$ was determined by the EDTA indirect titration method; HCO${}_{3}^{-}$ was determined using the double indicator neutralisation method. The detailed description of main soil physicochemical attributes is given in Table 1.

**Table 1:** Main physicochemical attributes of soil samples in the study area (mean ± S.D.).

Item	Unit	Value
Sand	%	75.31 ± 17.68
Silt	%	23.35 ± 16.63
Clay	%	1.34 ± 1.17
Main texture	/	Sandy Loam
EC	dS m^−1^	8.58 ± 12.02
pH	/	8.35 ± 0.42
K^+^	g kg^−1^	0.38 ± 0.04
Na^+^	g kg^−1^	18.30 ± 2.65
Ca^2+^	g kg^−1^	4.33 ± 0.46
Mg^2+^	g kg^−1^	0.43 ± 0.07
HCO${}_{3}^{-}$	g kg^−1^	2.13 ± 1.33
Cl^−^	g kg^−1^	6.25 ± 12.63
SO${}_{4}^{2-}$	g kg^−1^	13.98 ± 15.02

**Notes.**

S.D., indicates standard deviation.

### Spectral measurements under laboratory conditions and pretreatment

The VIS–NIR spectroscopic measurements in laboratory were conducted using a portable FieldSpec^®^3 ASD Spectroradiometer device with a resampling interval of approximately 1.0 nm in the measurement range (350–2,500 nm). The sampling intervals of the device are 1.4 nm and 2 nm, in the 350–1,000 nm range, and 1,000–2,500 nm range, respectively. Prior to spectrum measurements, each soil sample was placed into a petri dish in the dark (12 cm diameter, 1.8 cm depth) (Peng et al., 2014). The detailed measurement conditions of the step were given by Wang et al. (2017b). To avoid biased measurements, the spectrometer was corrected with a calibrated Spectralon^®^ panel with near 100% reflectance before the spectral measurement. Ten spectral curves were gathered, and then averaged as the final reflectance value. To minimize the effect of inherent spectral noise at the edges of spectra, the reflectance was reduced to 400–2,400 nm. Because of the slight fluctuations in the spectral reflectance, the Savitzky–Golay (S-G) algorithm was adopted in spectral smoothing and realized the two order polynomial fit in the window size of five data points (Savitzky & Golay, 1964). Subsequently, subjecting the spectra subset (400–2,400 nm range) to SG smoothing and absorbance (–lg R, R meaning the reflectance) processing. They comprised the data source of spectral analysis and model construction in this study and are provided as Table S1.

### Grünwald-Letnikov fractional derivative

The application of derivatives is an ideal treatment in spectral analysis (Viscarra Rossel, McGlynn & McBratney, 2006). The VIS–NIR reflectance was derivative-converted to reduce the influence of noise, amplify and reveal the greater spectral features (Wang et al., 2017a). The fractional derivative has expanded the theoretical concept of classic derivative, which has been successfully adopted in spectral data processing. To reduce the complexity of the discrete operation, the Grümwald–Letnikov definition of fractional derivative algorithm was employed for the calculations in the current study. A detailed description of the definitions is available in previous publications (Xia et al., 2017; Zhang & Chen, 2015). When the order is set as *α,* the *α*-order fractional derivative of function *f* (*x*) on the section of [ *β*, *γ*] is as follows:

(1) \begin{eqnarray*}{d}^{\alpha }f(x)=\lim _{h\rightarrow 0} \frac{1}{{h}^{\alpha }} \sum _{m=0}^{[(\mathrm{t}-a)/h]}(-1)^{m} \frac{\Gamma (\alpha +1)}{m{!}\Gamma (\alpha -m+1)} f(x-mh)\end{eqnarray*}

where *h* represents step length, and [(*γ* − *β*)∕*h*] represents integer part of (*γ* − *β*)∕*h*.

The Gamma function is defined as:

(2) \begin{eqnarray*}\Gamma (z)=\int \nolimits \nolimits _{0}^{\infty }\exp \nolimits (-u){u}^{z-1}du=(z-1){!}\end{eqnarray*}

Based on the actual resampling interval of the spectral sensor is 1 nm, then set *h* = 1, Eq. (1) can be written as follows:

(3) \begin{eqnarray*} \frac{{d}^{\alpha }f(x)}{d{x}^{\alpha }} \approx f(x)+(-\alpha )f(x-1)+ \frac{(-\alpha )(-\alpha +1)}{2} f(x-2)+\cdots \cdots \frac{\Gamma (-\alpha +1)}{n{!}\Gamma (-\alpha +n+1)} f(x-n).\end{eqnarray*}

Therefore, Eq. (3) can be considered as the specific formula for the calculation in this study. It is noted that the 0.0 order means that the data are not processed, which means the raw value (Hollkamp, Sen & Semperlotti, 2017). According to Eq. (3), set order interval as 0.1, fractional derivatives of reflectance and their absorbances, range from the 0.0 to the 2.0 order were calculated in the current study,

### Model calibration, evaluation, and comparison

The PLSR and RF were applied to establish models for soil salinity quantitative estimation from pretreated VIS–NIR reflectance (400–2,400 nm range). In this section, to ensure the full range of soil salinity is represented in both dataset, we used the Kennard-Stone (K-S) algorithm for samples selection (Wang et al., 2017b). The whole dataset (*n* = 400) was split into two sections: 80% for calibration (cross-validation, *n* = 320) and 20% for prediction (independent validation, *n* = 80).

#### Partial least squares regression (PLSR)

The PLSR has been frequently used in spectral quantitative research due to its superiority of dimension reduction and the synthesis. Detailed description of PLSR is available in Wold, Sjöström & Eriksson (2001). In the calculation procedure of the step, PLSR follows a linear multivariate model to associate the independent variables (*X*, reflectance in this research) and dependent variables (*Y*, soil salinity in this research) and select latent factors (variables). Thereby, it compresses *X* variables into a small number of latent variables (LVs) to maximize the covariance between the LV scores and *Y* variables. To identify the ideal number of LVs, leave-one-out cross validation (LOOCV) was conducted. Parameter optimization and modeling were implemented with the PLS_Toolbox (version 7.9) based on MATLAB^®^ software version R2012a (MathWorks, Inc., Natick, MA, USA).

#### Random forest (RF)

The RF, a recently prevalent machine learning method for classification and regression, can estimate complicated non-linear relationships between independent variables and response variables (Pal, 2005; Wang et al., 2018). It has proved a promising regression method especially in estimating soil attributes using VIS–NIR (Shi & Horvath, 2006). The RF aggregates various predictions based on changes in the training dataset through resampling. This algorithm consists of an ensemble of stochastic classification and regression trees (CART). Consequently, RFs are developed based on a combination of bagging method and randomized subspace method and then applied at each split in the tree. To grow each tree, the size of the variables subset *(mtry*) has to be selected by the user. Each decision tree grows until reaching a predefined minimum number of nodes (*nodesize*) on the new training dataset via random feature selection. In this research, the number of trees (*ntree*) was set to 500, both the size of the variables subset (*mtry*) and the minimum number of nodes (*nodesize*) were set to 2. The parameter selection and regression were conducted using Random Forest package (version 4.6-12) based on R software (version 3.4.0) (Douglas et al., 2018; Liaw, 2002).

#### Model evaluation and comparison

Two models, PLSR versus RF, were constructed and cross-validated with the training dataset, and independently validated via the testing dataset, separately. For the assessment of performance of spectroscopic models: (1) the coefficients of determination (*R*^2^), (2) root mean square errors (RMSE), and (3) ratio of performance to deviation (RPD) were calculated and compared individually. The definitions and formulae for the indices were given by Shi et al. (2014).

Based on the estimating model classification criterion illustrated by Viscarra Rossel et al. (2006), the inversion models could be partitioned into six classifications: Category A (RPD ≥ 2.50) is the excellent model; Category B (2.00 ≤*RPD* < 2.50) is the very good quantitative model; Category C (1.80 ≤*RPD* < 2.00) is the good model, the quantitative estimation might be possible; Category D (1.40 < *RPD* ≤ 1.80) is the fair model with limited performance; Category E (1.00 < *RPD* ≤ 1.40) is the poor model, which could only distinguish the difference of the high and low levels; Category F (*RPD* ≤ 1.00) is the unreliable model. Generally, optimal models with highest *R*^2^ (approach to 1) and RPD but lowest RMSE (approach to 0) would be selected.

**Figure 2:**
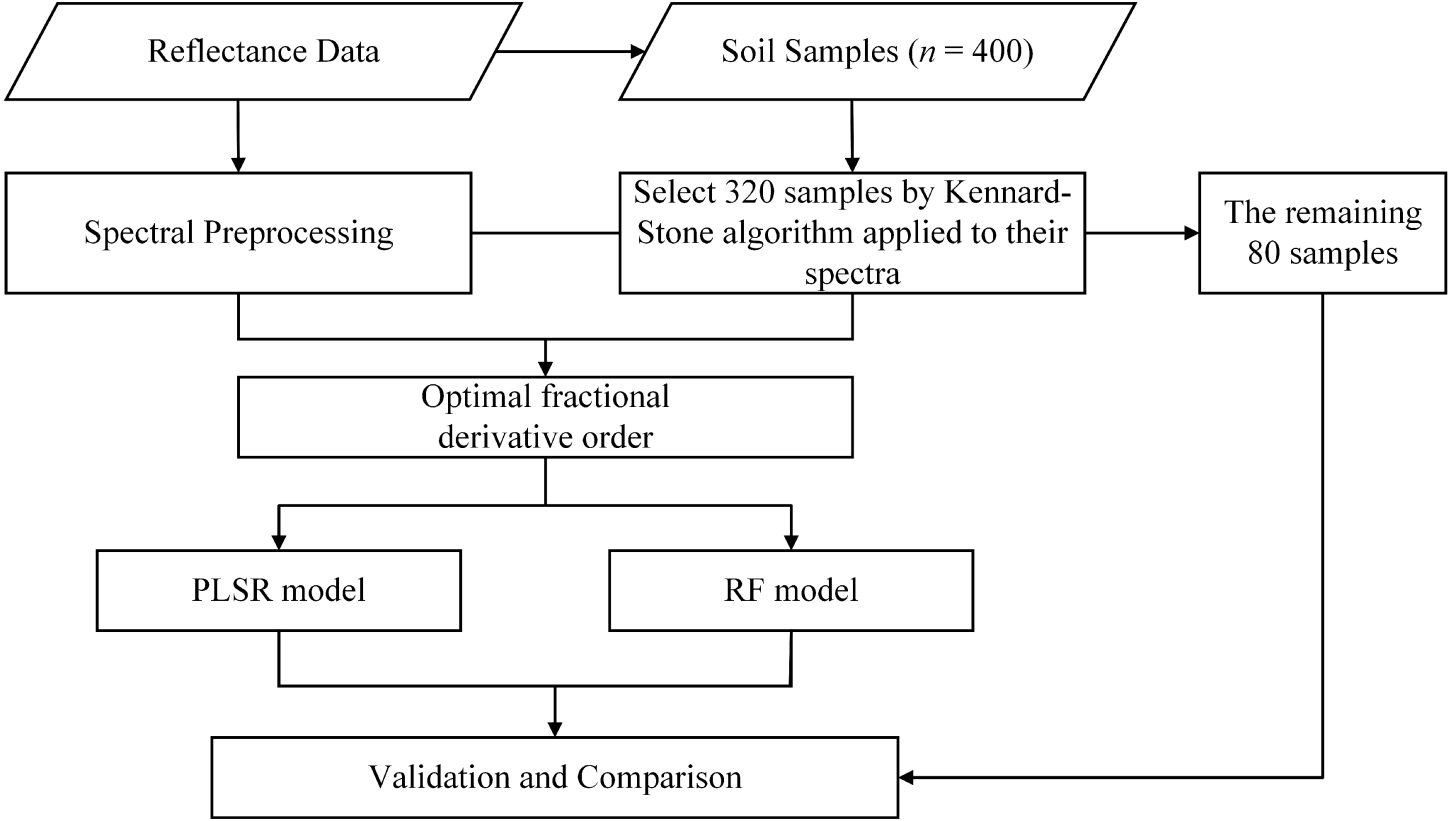
Flow chart of the study.

The steps of model construction and validation are illustrated in Fig. 2.

## Results

### Descriptive statistical analysis

**Figure 3:**
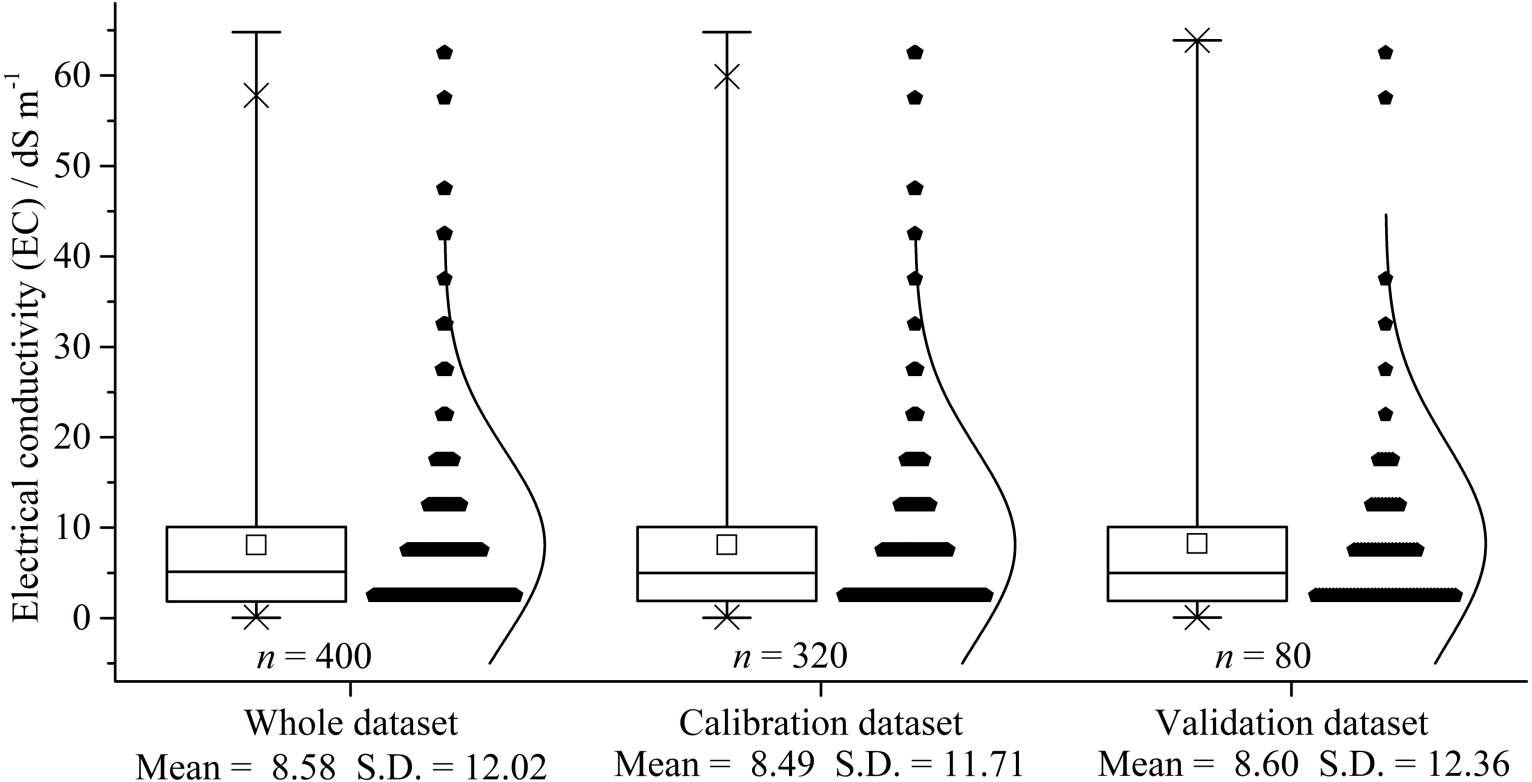
Box plot and distribution of soil salinity for the whole, calibration, and validation dataset (dS m ^−1^). S.D. indicates standard deviation.

The soil salinity of the study area varied widely between 0.03 and 64.80 dS m^−1^, with an average salinity of 8.58 dS m^−1^, standard deviation of 12.02 dS m^−1^, and a high coefficient of variation of 140.87% (>100%) (Fig. 3). The relative high mean salinity indicated that the surface soils were salt-affected in the Ebinur Lake wetland. Compared to the range of the salinity (0.03–64.80 dS m^−1^) for the calibration dataset, the validation dataset had a similar range of 0.06–63.42 dS m^−1^ with mean and standard deviation of 8.60 dS m^−1^ and 12.36 dS m^−1^, respectively. The results showed that the distribution of the soil salinity of all datasets was left-skewed in contrast to the standardized normal distribution. The statistical results of soil salinity in both calibration and validation dataset were similar to those of the whole dataset; consequently, the soil salinity of both datasets adequately represent the entire dataset.

### Reflectance of different soil salinity

**Figure 4:**
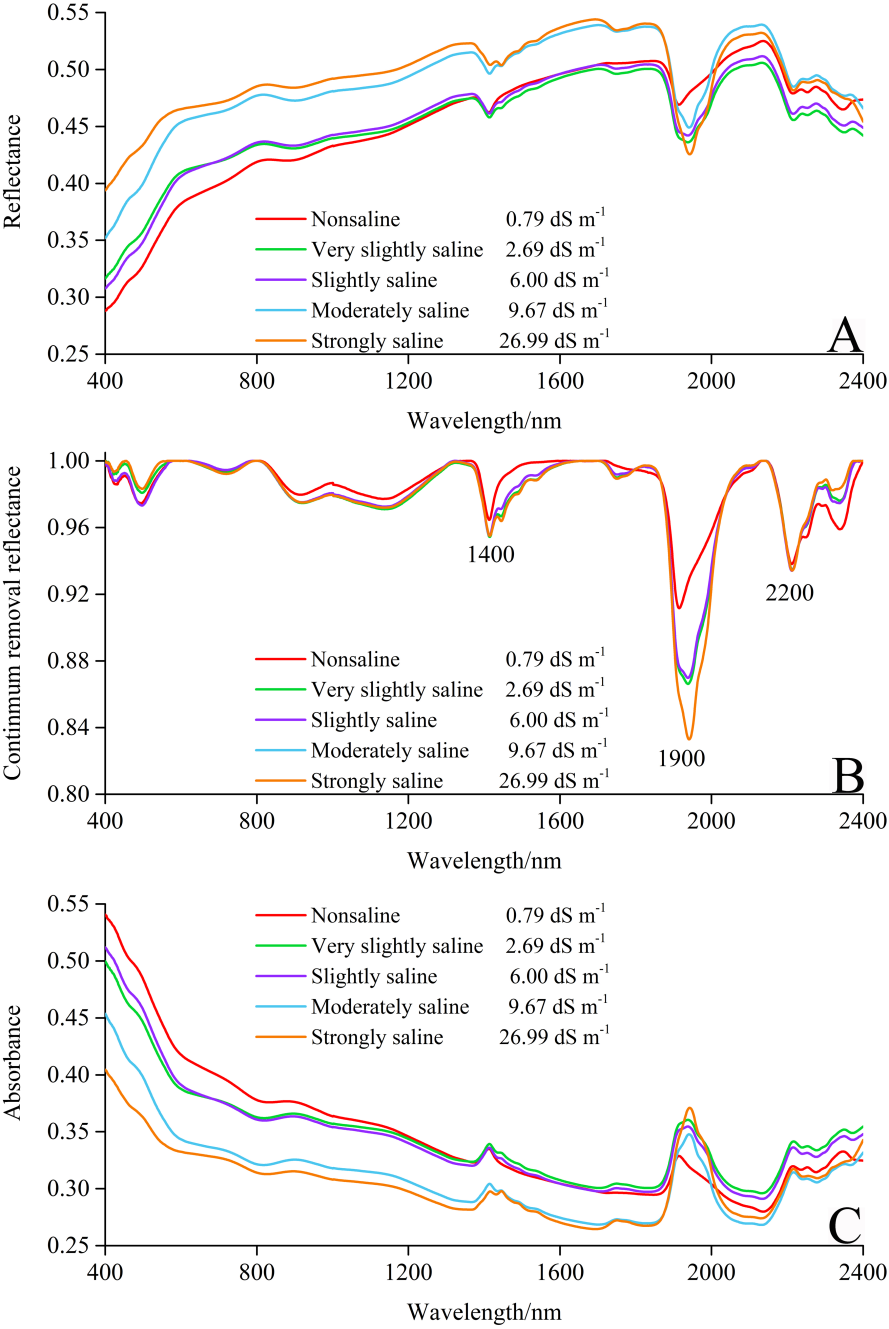
Reflectance spectra curves of soils with different salinity degrees. (A) Spectral curves. (B) Continuum removal curves. (C) Absorbance curves.

Based on the standard of different soil salinity level outlined by the United States Department of Agriculture (USDA), all 400 samples were classified into five different classes of soil salinity: non-saline (0–2 dS m^−1^), very slightly saline (2–4 dS m^−1^), slightly saline (4–8 dS m^−1^), moderately saline (8–16 dS m^−1^), and strongly saline (>16 dS m^−1^) (Schoeneberger et al., 2002; Shahid & Rehman, 2011). The soil reflectivity and spectral features vary with the different level of soil salinity (Fig. 4A). As seen in the diagram, spectral curves of soil with different salinity followed a similar shape. Notably, there were significant differences between moderately saline, strongly saline, and the other three degrees of soil salinity.

The continuum removal (CR) technique ordinarily can restrain the noise of background and emphasize weak absorption features in the spectra (Ji et al., 2014). The corresponding CR reflectance are illustrated in Fig. 4B. The three main absorption features were located at around 1,400, 1,900, and 2,200 nm, respectively. The absorption features at 1,400 nm are a representative absorption region for water combined with the bending and stretching vibration of the O-H bonds of free water (Shi et al., 2014). The regions near 1,900, and 2,200 nm in the combination range exist due to the bending and stretching vibrations of Al–OH and Mg–OH, respectively (Pu et al., 2003; Viscarra Rossel, McGlynn & McBratney, 2006a). Considering the essence of absorbance (−lg R), the absorbance curves are the reversal of spectral curves (Figs. 4A and 4C).

### Influence of spectral preprocessing methods

In the current study, all spectral reflectance data and according absorbances preprocessed by the fractional derivative were used for the model construction. Various fractional derivative orders had significant effects on the outcomes of soil salinity estimating models (Table 2). Compared with the PLSR models based on 0.0 order (without pretreatment of fractional derivative), besides the 1.4 order of absorbance, 1.5–1.8 orders of reflectance and absorbance improved the accuracies (RPD ≥ 2.00). The model based on the 1.5 order of absorbance possessed the optimum estimation performance (*R*^2^ = 0.87, RMSE = 5.23 dS m^−1^, and RPD = 2.40). In contrast, the pretreatment of 0.5 order fractional derivative resulted in the least acceptable results (*R*^2^ = 0.54 and 0.52, RMSE = 10.08 dS m^−1^ and 10.27 dS m^−1^, and RPD = 1.19 and 1.17 for reflectance and absorbance models, respectively). Excluding it, the PLSR models built on fractional derivative outperformed those using the classic integer derivatives (FD and SD). However, the parameters did show gradual improvement with the increase from the order 1.0 to 1.5. With increasing order, the RMSE and RPD values of the models gradually decreased. As the order increased to 1.5, the performance of the model improved drastically (Table 2). Thereby, the calibrations of the eight spectral methods achieved desirable performances with PLSR (1.5-order, 1.6-order, 1.7-order, and 1.8-order based on reflectance, and 1.5-order, 1.6-order, 1.7-order, and 1.8-order based on absorbance, respectively) and were used for the model construction.

**Table 2:** Results of leave-one-out cross validation for PLSR of both reflectance and absorbance treated by fractional derivatives.

Order	Reflectance				Absorbance			
	Latent variables	*R*^2^	RMSE (dS m^−1^)	RPD	Latent variables	*R*^2^	RMSE (dS m^−1^)	RPD
0.0	4	0.64	9.27	1.30	3	0.58	9.80	1.23
0.1	4	0.66	9.07	1.33	4	0.63	9.37	1.29
0.2	4	0.69	8.75	1.38	4	0.68	8.90	1.36
0.3	4	0.71	8.56	1.42	4	0.67	8.98	1.35
0.4	5	0.77	7.77	1.57	4	0.68	8.90	1.36
0.5	2	0.54	10.08	1.19	2	0.52	10.27	1.17
0.6	2	0.56	9.93	1.21	2	0.55	10.03	1.20
0.7	5	0.81	7.20	1.70	3	0.67	8.97	1.35
0.8	3	0.71	8.49	1.43	3	0.66	9.04	1.34
0.9	3	0.72	8.43	1.44	3	0.68	8.87	1.36
1.0	3	0.70	8.70	1.39	3	0.66	9.05	1.34
1.1	5	0.84	6.63	1.86	5	0.79	7.43	1.65
1.2	5	0.84	6.76	1.82	5	0.82	7.03	1.75
1.3	5	0.84	6.73	1.83	5	0.84	6.68	1.84
1.4	5	0.84	6.36	1.94	5	0.84	6.18	1.99
1.5	5	0.84	6.18	2.01	5	0.87	5.23	2.40
1.6	5	0.86	6.00	2.07	5	0.84	6.06	2.05
1.7	5	0.85	6.16	2.02	5	0.84	6.07	2.04
1.8	5	0.84	6.19	2.00	5	0.83	6.09	2.03
1.9	5	0.84	6.73	1.83	5	0.83	6.85	1.79
2.0	5	0.83	6.85	1.79	5	0.83	6.98	1.76

### Performance of PLSR and RF models for salinity quantitative estimation

**Figure 5:**
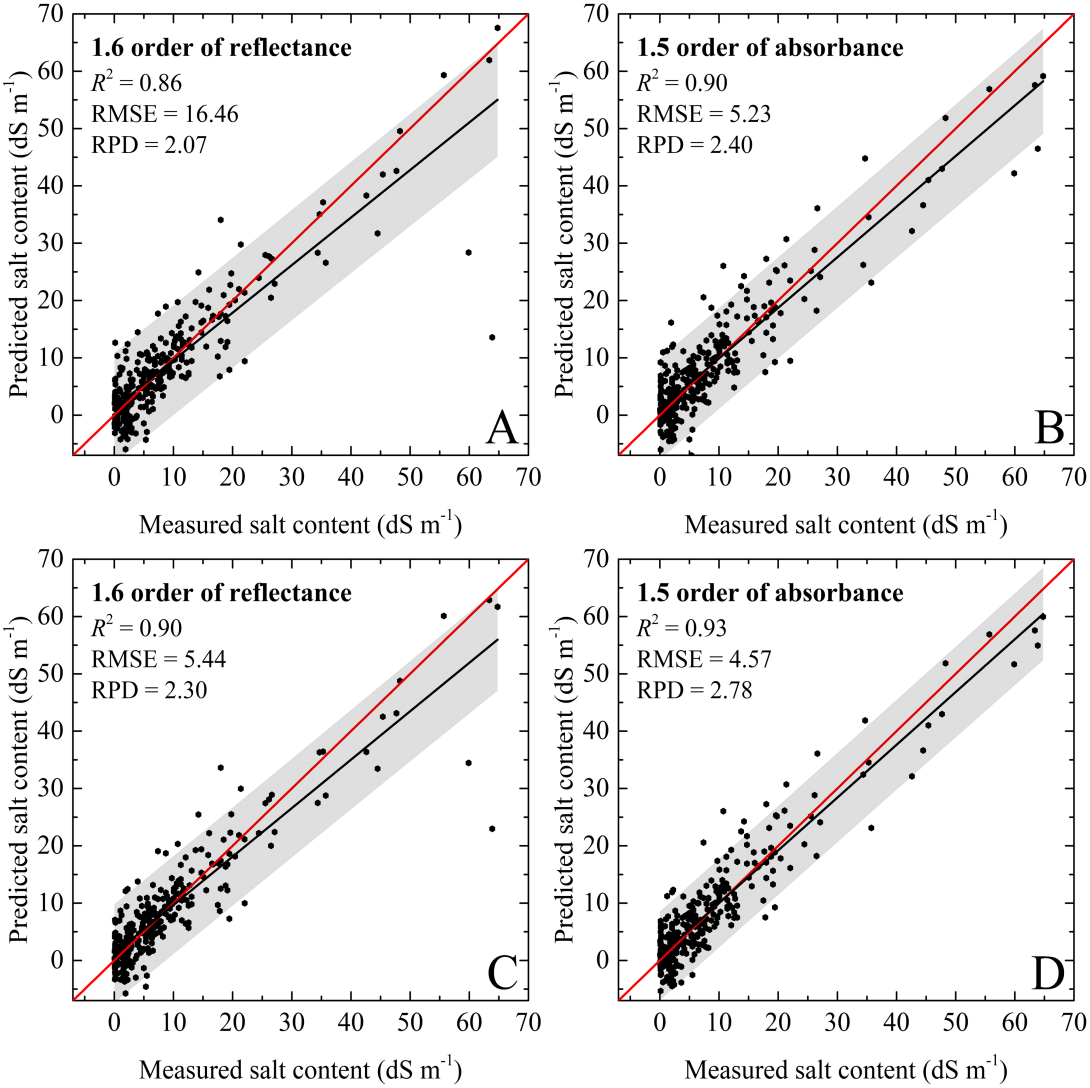
The soil salinity quantitative models using calibration dataset. (A) PLSR model based on 1.6 order of reflectance. (B) PLSR model based on 1.5 order of absorbance. (C) RF model based on 1.6 order of reflectance. (D) RF model based on 1.5 order of absorbance. The black line represents the fitted line, the red line represents the 1:1 line, and the gray regions represent the confidence intervals with 95% probability.

In this research, different calibration methods produced various estimation accuracies for soil salinity. For the calibration dataset of PLSR, the predicting model based on the absorbance (1.5 order) had the best performance (${R}_{C}^{2}=0.90$, RMSE_*C*_ = 5.23 dS m^−1^, and RPD_C_ = 2.40), while the worst results were with the 1.8 order of reflectance (${R}_{C}^{2}=0.85$, RMSE_*C*_ = 6.19 dS m^−1^, and RPD_*C*_ = 2.00). Except for the 1.6 order, PLSR models based on the fractional derivative orders of absorbance outperformed the according reflectance models at the same order (Figs. 5A and 5B). Compared to PLSR, the RF models had better performances than the PLSR models with each preprocessing technique, and RPD ranged from 2.00 to 2.78. The RF model based on the absorbance (1.5 order) possessed the best capability (${R}_{C}^{2}=0.93$, RMSE_*C*_ = 4.57 dS m^−1^, and RPD_*C*_ = 2.78), followed by the model based on the 1.6 order. In addition, all calibration models had very good RPD values exceeding 2.00 for the above eight spectral pre-processing methods (Tables 3 and 4; Figs. 5C and 5D).

**Figure 6:**
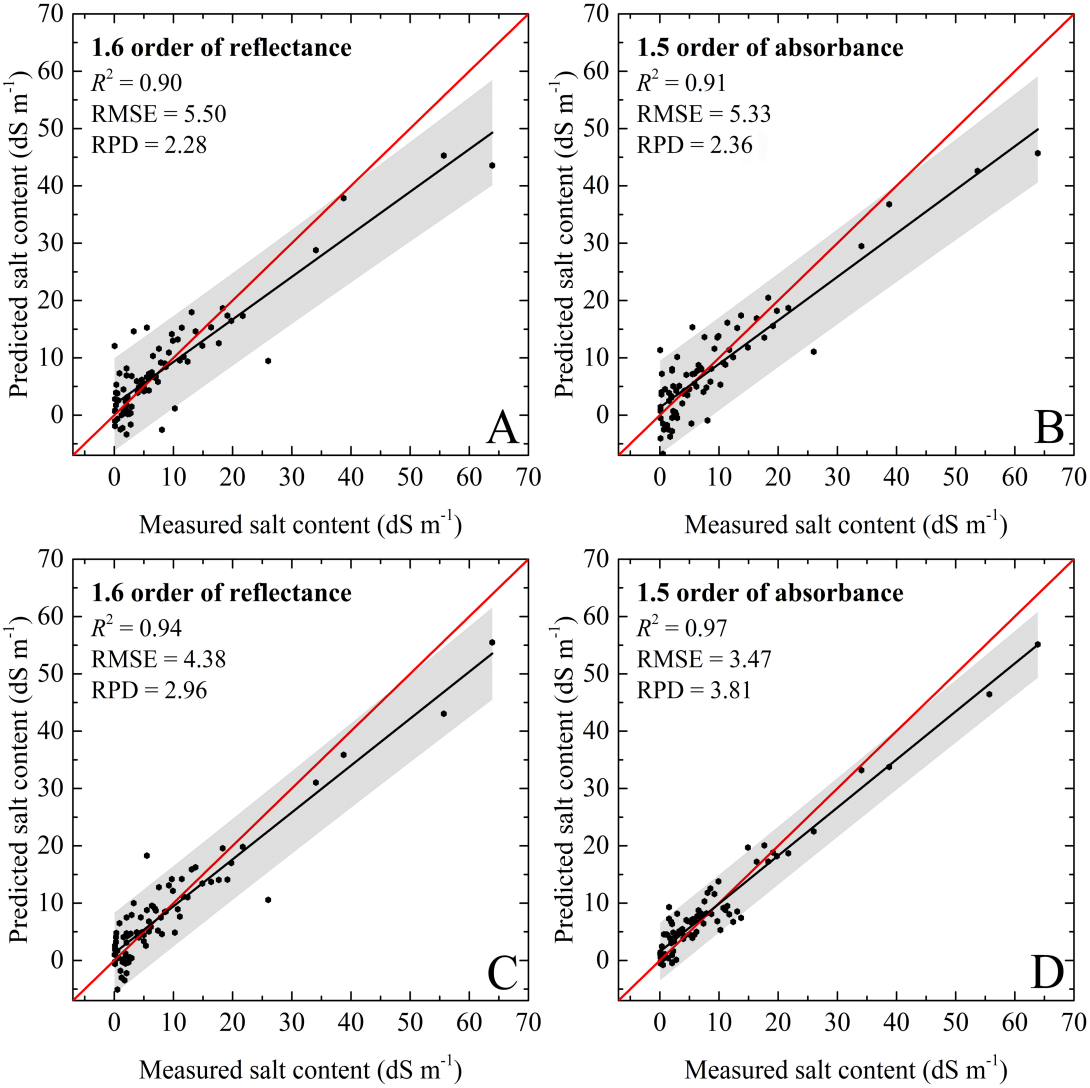
The soil salinity quantitative models using validation dataset. (A) PLSR model based on 1.6 order of reflectance. (B) PLSR model based on 1.5 order of absorbance. (C) RF model based on 1.6 order of reflectance. (D) RF model based on 1.5 order of absorbance. The black line represents the fitted line, the red line represents the 1:1 line, and the gray regions represent the confidence intervals with 95% probability.

With respect to the validation dataset, it had similar variation trends compared to those of the calibration dataset, but had higher prediction accuracies. For the PLSR model, the validation results with the 1.5 order of absorbance was most accurate (${R}_{V}^{2}=0.91$, RMSE_*V*_ = 5.33 dS m^−1^, and RPD_*V*_ = 2.36). The RF models with all spectral preprocessing produced good performance, and the RPD values were close to or even greater than 2.50. The RF models with the 1.5 order of absorbance showed excellent performance (${R}_{V}^{2}=0.97$, RMSE_*V*_ = 3.47, and RPD_*V*_ = 3.81 ≥ 2.50). The validation accuracies of PLSR models were slightly lower than those of RF, but still very good for the soil salinity quantitative estimation (${R}_{V}^{2}=0.89$–0.91, RMSE_*V*_ = 5.33–5.89 dS m^−1^, and RPD_*V*_ = 2.19–2.36). For the validation dataset, the slopes for the PLSR and RF models based on 1.6 order of absorbance were well distributed to the 1:1 line which indicated excellent validations. However, the slopes for the PLSR and RF models based on 1.5 order of reflectance were under the 1:1 line, and the data points were relatively discrete (Tables 3 and 4; Fig. 6). In addition, some negative values were recorded in the prediction results.

**Table 3:** The cross validation of the calibration dataset (*n* = 320) and the capability of the validation dataset (*n* = 80) for the quantitative estimation of soil salinity using PLSR model with different spectral types.

Spectral types	Order	Calibration dataset			Validation dataset		
		*R*^2^	RMSE (dS m^−1^)	RPD	*R*^2^	RMSE (dS m^−1^)	RPD
Reflectance	1.5	0.86	6.18	2.00	0.90	5.63	2.22
	1.6	0.86	6.00	2.07	0.90	5.50	2.28
	1.7	0.85	6.16	2.01	0.90	5.60	2.24
	1.8	0.85	6.19	2.00	0.89	5.89	2.12
Absorbance	1.5	0.90	5.23	2.40	0.91	5.33	2.36
	1.6	0.84	6.06	2.05	0.91	5.36	2.35
	1.7	0.84	6.07	2.04	0.90	5.44	2.31
	1.8	0.85	6.09	2.03	0.89	5.71	2.19

**Table 4:** The cross validation of the calibration dataset (*n* = 320) and the capability of the validation dataset (*n* = 80) for the quantitative estimation of soil salinity using RF model with different spectral types.

Spectral types	Order	Calibration dataset			Validation dataset		
		*R*^2^	RMSE (dS m^−1^)	RPD	*R*^2^	RMSE (dS m^−1^)	RPD
Reflectance	1.5	0.87	6.11	2.00	0.90	5.57	2.26
	1.6	0.90	5.44	2.30	0.94	4.38	2.96
	1.7	0.90	5.57	2.24	0.91	5.41	2.34
	1.8	0.89	5.63	2.22	0.90	5.58	2.26
Absorbance	1.5	0.93	4.57	2.78	0.97	3.47	3.81
	1.6	0.90	5.42	2.30	0.93	4.72	2.71
	1.7	0.90	5.49	2.26	0.92	5.10	2.49
	1.8	0.88	5.55	2.25	0.91	5.23	2.42

## Discussion

### Fractional derivative results of the reflectance

**Figure 7:**
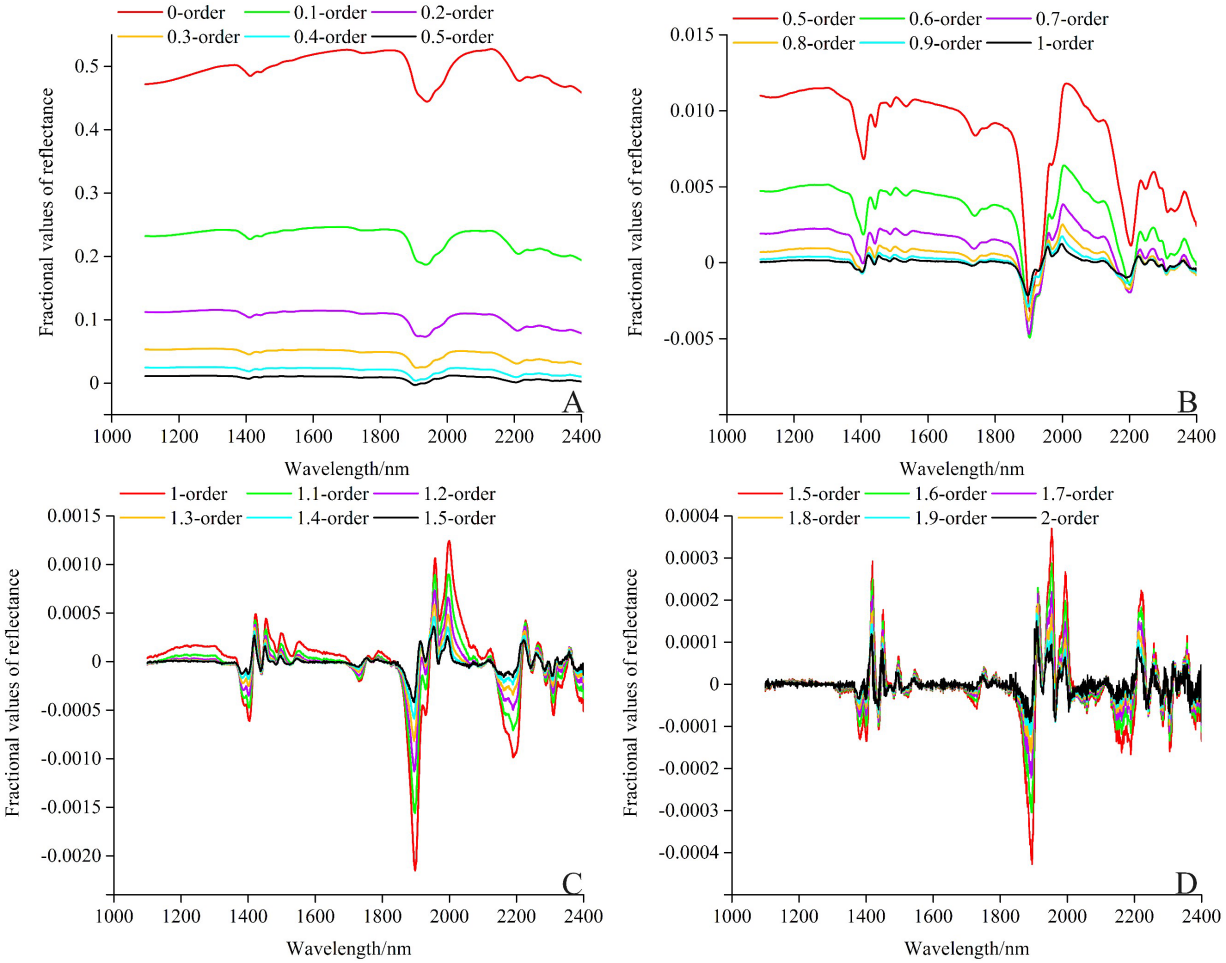
Fractional derivative results of the reflectance in the range of LW–NIR (1,100–2,400 nm). (A) 0–0.5 order. (B) 0.5–1.0 order. (C) 1.0–1.5 order. (D) 1.5–2.0 order.

Fractional order derivative processing influences the spectral data to a certain degree (Schmitt, 1998). The fractional derivative results of the average reflectance in the range of 1,100–2,400 nm (long-wavelength near-infrared spectroscopy, LW–NIR) are illustrated in Fig. 7. With the order increasing from 0.0 to 1.0, the fractional derivative curves slowly followed the FD (1.0 order) curve, and became sensitive to the slope and less sensitive to reflectance. From 1.0 to 2.0, the fractional derivative curves slowly approached the SD (2.0 order) curve and, to a certain extent, became more sensitive to the curvature and less sensitive to the slope (Wang et al., 2017a). The fractional derivative results of the reflectance showed a fluctuating trend in this region. Some less obvious absorption peak information was magnified. The strengthening of peak intensity in VIS–NIR was very important to the further exploration of its reflection mechanism (Li et al., 2014). Compared with the conventional raw reflectance, FD, and SD, more spectral characteristics were refined of the spectrum reflectance treated by the pretreatment of fractional derivative, and are provided as Table S2.

### Effects of fractional derivative on estimation models

Due to the abundant spectral information and the rapid data acquisition, VIS–NIR has been frequently used for assessing multiple soil parameters. To obtain more spectral information and features and to further improve the robustness and capability of the models, it is vital to preprocess raw reflectance (Nawar et al., 2016). Spectral derivative analysis is a simple and effective preprocessing method which is commonly used for the enhancement of spectral information. In general, the order interval is set to 1.0, and the regression models are constructed based on the FD or SD. However, pretreatment of the FD and SD might cause the loss of spectral information (Wang et al., 2017a). In this research, raw reflectance and absorbance without pretreatment (0.0 order) and the corresponding FD and SD were applied for the model construction as well. For reflectance, the PLSR model based on 0.0 order is poor with the lower RPD (1.30 ≤ 1.40). Once the order reached 1.0 (FD), the quantifying capability slightly improved; however, it was not suitable for the quantitative estimation of soil salinity (*R*^2^ = 0.70, RMSE = 8.70 dS m^−1^, and RPD = 1.39 ≤ 1.40). With regard to SD, the corresponding model with a value of RPD = 1.79 ≤ 2.00 was better than the models based on FD, and still retained an inadequate prediction ability; hence, it was unsuitable for quantitative estimation. In the current study, the various fractional derivative orders significantly affected the results of soil salinity calibration models (Table 2). In addition, the variation trend of the precision parameters was obvious; where the model was based on the 0.5 order, it showed the worst performance with the lowest RPD (1.19) and the highest RMSE (10.08 dS m^−1^). In comparison, the accuracies of the calibrition models based on absorbance were slightly weaker than those of the reflcetance models, while the condition of the validation data sets showed an inverse pattern. The preprocessing of the 1.6 order of absorbance obtained the best performance followed by the 1.5 order of reflectance.

Our results showed that the 1.5 order of absorbance was the optimal fractional derivative order for PLSR and RF based estimation of soil salinity. The pretreatment of fractional derivative orders has also been applied in previous research to model various soil properties (Wang et al., 2017a; Zhang et al., 2016a). For example, Wang et al. (2017b) applied the fractional derivative algorithm for the pretreatment of the reflectance of soil, and the PLSR results were effectively improved.

Compared to the common integer derivative (FD and FD), the preprocessing of fractional derivative with a narrower order interval could collect more details and features from spectra and further lay the foundation for the improvement of the capability of the predecting models.

### Comparison between PLSR and RF models in estimating soil salinity

In the current study, the PLSR and RF were applied for the quantitative estimation of soil salinity of the Ebinur Lake wetland. The two techniques showed different accuracies depending on the different type of reflectance. Between the two calibration methods, RF was statistically superior to PLSR, while PLSR provided slightly weaker predictive power. Compared with existing results obtained using PLSR (*R*^2^ = 0.66–0.87) and RF (*R*^2^ = 0.78–0.91), the soil parameters models developed in the current study could be regarded as acceptable under the classification standard (Islam, Singh & McBratney, 2003; Nawar et al., 2014; Shepherd & Walsh, 2002; Shi et al., 2014; Wang et al., 2018; Wang et al., 2017b; Zhang et al., 2016a). The PLSR technique could effectively solve multiple collinearity problems among independent variables, but only simulate the potential linear relationship between some specific soil attributes and corresponding VIS–NIR reflectance. In reality, the distribution of soil properties is mostly skewed distribution rather than the standardized normal distribution, and the application of linear regression method such as PLSR may be insufficient. Thereby, the RF model typically yields superior estimation accuracies if a non-linear relationship exists between predictor and response variables.

RF recorded the excellent validation accuracies based on the effective preprocessing method with ideal *R*^2^ (0.90–0.97), RMSE_*V*_ (3.47–5.57 dS m^−1^), and all RPD greater than 2.00 (Table 4). Compared to the validation accuracy, the performance of calibration was slightly lower but still acceptable (*R*^2^ between 0.87 and 0.93, RMSE_*V*_ between 4.57–6.11 dS m^−1^), RPD between 2.00 and 2.78). In terms of RPD, the best RF model was calibrated with the 1.5 order of absorbance, and the optimal model performance was obtained (*R*^2^ = 0.93 and RMSE_*C*_ = 4.57 dS m^−1^). For the validation dataset, the PLSR model with the same pretreatment method performed relatively well across the seven models except in the case of the 1.5 order of absorbance model (RMSE_*V*_ = 5.23 dS m^−1^, RPD = 2.40; Table 3). However, the calibration dataset of soil salinity had an extremely wide range, varying from 0.03 to 64.80 dS m ^−1^ with a standard deviation value of 12.10 dS m^−1^, and included some samples of non-saline soil. The high accuracies of PLSR and RF with validation dataset might be attributed to the data distribution (88.750% of soil samples were saliferous). Zhang et al. (2016a) set the order interval to 0.2 and indicated that the model constructed by 250 feature bands based on 1.2-order derivative of absorbance possessed an excellent capacity of estimating soil salinity. Generally, the pretreatment of fractional derivative could refine and enhance the spectral characteristics of the spectrum reflectance (Wang et al., 2017c). Compared with the previous studies, the combination of RF and narrower fractional order interval could significantly improve the estimations accuracies and generalization ability.

### Research limitations

The superior performance of RF in comparison with the PLSR models tested could be explained by its outstanding ability to deal with the non-linear pattern and generate precise estimation, which has been reported in the previous research of quantifying soil properties via VIS–NIR (Morellos et al., 2016; Stenberg et al., 2010; Viscarra Rossel & Behrens, 2010). Results of the current study were in accord with this research. The machine learning algorithm with more parameters or hyper-parameters often requires massive complex training, though it records better accuracy. An ideal algorithm should exhibit high simulation precision and also include simple trained parameters and training time consumption. With respect to the machine learning algorithm, the training approximation and generalization of the generated models are strongly sensitive to the calibration dataset (Stallkamp et al., 2012). Strong interpretability is also vital to the algorithm. For the detection of target content, the capability of Multilayer feed-forward neural network (MLFN) has been examined, which has proved a simple automatic method with good forecasting precision (Yang et al., 2014). We will use more unsupervised and semi-supervised learning algorithms (e.g., Principal component analysis and K-means clustering) to identify and eliminate abnormal samples. The synthesis of different algorithms should be further tested to verify their capability for soil salinity quantitative estimation in a larger scale in further research.

In this study, the order interval (0.1) was not sufficiently fine and the 1.5 order and the 1.6 order seemed to represent a critical point. A self-adapting algorithm of order selection of fractional derivatives is currently being conducted. Thus, smaller order intervals could be obtained. The application of remote sensing data for mapping soil parameters depends on the different spectral behavior, spatial–temporal distribution of soils, and the vegetation on the terrain surface. There are many strong signals in the range between 1,900–2,200 nm. Furthermore, the salinity is not a unique factor of forming soil reflectance properties. The VIS–NIR predicting performances of soil salinity might be affected due to the fact that adsorption properties of soluble salts in these electromagnetic ranges are weaker than those of water, soil iron, organic matter, certain types of clay minerals, and some other soil components. To further improve the prediction accuracy, the most dominant factor of soil reflectance with different salinity degrees will be analyzed in the future study. The fractional derivative has not been tested on the remote sensing data collected from different platforms, e.g., Landsat, Hyperion, and unmanned aerial vehicle (UAV). Therefore, taking into account the soil sampling depth, the salt/soil composition, the soil moisture content, and some other factors, further research should focus on the possible combination of satellite imagery, field-, and laboratory-derived spectra data.

## Conclusions

In this study, soil salinity was measured under laboratory conditions according to the spectral reflectance of 400 soil samples from the Ebinur Lake wetland. The fractional derivative was introduced to the pretreatment of spectral data to obtain a robust quantitative prediction model. Fractional derivative results of the reflectance showed a fluctuating trend in the range of LW–NIR. Some less obvious absorption peak information was magnified to a certain extent. More spectral characteristics were refined by the spectrum reflectance treated by fractional derivative. The 1.5 order and the 1.6 order were the most important fractional derivative orders for the soil salinity quantitative estimation. Both in the calibration dataset and validation dataset, RF models performed better than PLSR models. Among these established models, the most effective model was established based on RF with the 1.5 order derivative of absorbance, with the optimal values of *R*^2^(0.93), RMSE (4.57 dS m^−1^), and RPD (2.78 ≥ 2.50). This model showed an excellent predictive performance of estimating soil salinity of the Ebinur Lake wetland. The pretreatment of fractional derivative could flourish the spectra processing technology. Such an approach could be useful for monitoring multiple land surface parameters with higher accuracy.

##  Supplemental Information

10.7717/peerj.4703/supp-1 Table S1 Raw data: Reflectance of soil samples (*n* = 400)

10.7717/peerj.4703/supp-2 Table S2 The fractional derivative results of the average reflectance in the range of 1,100–2,400 nm

## Additional Information and Declarations

### Data Availability

The following information was supplied regarding data availability: The raw data are provided in the Supplemental Files.
